# Everybody's Page

**Published:** 1889-09-14

**Authors:** 


### Everybody's Page.

During the fourteen years, 1870 to 1883, there were in England and Wales 635 deaths from hydrophobia, with a minimum of 36 in 1870 and a maximum of 82 in 1877.

Here is a true case of treatment by suggestion. Anxious mamma: "Little Dick is upstairs, crying with the toothache." Practical papa: "Take him round to the dentist's." "I haven't any money." "You won't need any money. The toothache will stop before you get there."

A familiar nuisance in Australia is the Cape weed, which, by its resemblance in the distance to buttercups, recalls an English spring, but which has a very different and destructive effect upon the herbage in which it grows. This weed is said to have emanated from the Melbourne Botanical Gardens, to which it was introduced about thirty years ago.

Parents and guardians of very young children cannot be too often or too strongly reminded that Swiss milk is not a proper food for infants, however useful it may be found by adults as a substitute for cow's milk. Inquests are constantly held on emaciated babies who have been fed on Swiss milk, and it has not properly nourished them. Failing mother's milk, a baby should have cow's milk.

It has long been held that differences of weight in atmospheric pressure have a marked effect on chest complaints, but it has been reserved for Dr. Potain, of the Paris Faculty of Medicine, to make an experiment in support of the theory on the most practical scale. He ascended in a captive balloon to a height several hundred feet higher than the Eiffel Tower, taking with him five medical students and a patient whose chest is weak. The object of the expedition was to test the effect on the patient of the density and temperature of these regions of the upper air.

The housing of the poor is a question which agitated Charles Dickens greatly. In the "Old Curiosity Shop" he raised a bitter cry against overcrowding: "Oh! if those who rule the destinies of nations would but remember this—if they would but think how hard it is for the very poor to have engendered in their hearts that love of home from which all domestic virtues spring, when they live in dense and squalid masses where social decency is lost, or rather never found—if they would but turn aside from the wide thoroughfares and great houses, and strive to improve the wretched dwellings in byeways where only Poverty may walk, many low roofs would point more truly to the sky than the loftiest steeple that now rears proudly up from the midst of guilt and crime and horrible disease, to mock them by its contrast."

One of the most curious of our ancient charities is that of the Biddenden Maids. These two unfortunate women were born at Biddenden, in Kent, in the year 1800, joined to each other at the hips and shoulders. Their names were Eliza and Mary Chulkhurst, and they lived for thirty-four years. Then one fell ill and died, and the survivor was advised to be separated from her dead sister by dissection, but she refused, saying: "As we came together we will also go together." Within six hours she too was taken ill, and died also. By their will they bequeathed to the churchwardens of Biddenden a piece of ground, which now brings in a rental of forty guineas. This is expended in the purchase of five hundred quartern loaves, and cheese in proportion, which are distributed among the poor of the parish, and on Easter Sunday 1,000 small rolls, shaped like a gravestone, and bearing a bas-relief portrait of the unfortunate maids, are distributed to all who attend divine service.

The colonies are almost entirely wanting in special hospitals, and no other town in the world can boast so many hospitals as London. We are not quite sure it is a boast to be proud of.

The hot air treatment of phthisis does not seem very satisfactory. In a few cases it gives relief; but in many the published records of treatment seem to indicate fever, nausea, and pain as frequent results.

Natural gas, which was discovered in Pennsylvania, and was first used in the town of Pittsburg in 1883, has not proved an unmixed blessing. It is a cheap fuel and light, but it made the air too dry to breathe, and ruined furniture by cracking the joints. It had also an inconvenient habit of exploding, and it was not till experience and experiment had taught the proper apparatus and method of using it that it became a commodity of really practical value.

Snoring is not exactly a disease, and those who suffer from it endure it with great equanimity. So do not those who have to sleep—or lie awake against their will—within earshot of the snorers. Recently a request for advice as to a cure for snoring appeared in a medical journal. One facetious correspondent, in reply, said: "Bite the nose gently but firmly." Another, and more practical advice, is to tie a band or handkerchief over the mouth, thus forcing the snorer to breathe through his nose.

A warning to opium-eaters may be drawn from a case recently brought before a London magistrate. A soldier, aged 31, gave himself up on the charge of having murdered a man in Australia twelve years ago. Enquiries were made, which proved that the man who was supposed to be murdered was alive after the time given as the date of the crime, and then it transpired that the self-accused murderer was an opium-eater and subject to hallucinations. Consequently he was liberated, but it would be awkward to make such statements with regard to oneself before a tribunal that was ready to accept one's word on the subject.

An anecdote which we lately heard indicates to what lengths the age's passion for experiment will go. A boy having recovered from rheumatic fever, the doctor told his mother that she must be very careful with regard to him, as his heart was affected. The mother pondered over this and did not wholly believe it. Finally, she remembered having been told that a Turkish bath was dangerous for anyone suffering from heart disease, so she took the boy to have one just to see how it would affect him. Fortunately it did him no harm; so now she goes about boasting how much cleverer she is than the doctor. But if the boy had died, what then?

Of all the experiments made by vivisectors, the rat's tail one appears among the most useless. The late M. Paul Bert was the inventor of it. He sewed the tip of a rat's tail into the animal's back. After it had taken root in the new position he cut off the tail at the original root, and let Mr. Rat carry his tail upside down. After a time it was found that the reversed tail was sensitive, the inference being that the nerves of sensation can carry impulse either way. Recently, however, Dr. Koch has performed the same experiment on forty rats; but he finds that though the reversed tails adhere well enough they have no sensation. So nothing has been gained by the mutilation, and meanwhile—the poor rats! Will they not say "Let me fall into the teeth of the terrier and not into the hands of the scientist!"

